# PR interval prolongation in coronary patients or risk equivalent: excess risk of ischemic stroke and vascular pathophysiological insights

**DOI:** 10.1186/s12872-017-0667-2

**Published:** 2017-08-24

**Authors:** Yap-Hang Chan, Jo Jo Hai, Kui-Kai Lau, Sheung-Wai Li, Chu-Pak Lau, Chung-Wah Siu, Kai-Hang Yiu, Hung-Fat Tse

**Affiliations:** 1 Division of Cardiology, Department of Medicine, Queen Mary Hospital, University of Hong Kong, Rm 1928, Block K, Hong Kong, China; 2 Division of Neurology, University of Hong Kong, Hong Kong, China; 3 Department of Medicine, Tung Wah Hospital, Hong Kong, SAR China; 4 Research Center of Heart, Brain, Hormone and Healthy Ageing, University of Hong Kong, Hong Kong, China

**Keywords:** PR interval prolongation, Cardiovascular death, Myocardial infarction, Ischemic stroke, Carotid intima-media thickness, Vascular function, Pathophysiological mechanism

## Abstract

**Background:**

Whether PR prolongation independently predicts new-onset ischemic events of myocardial infarction and stroke was unclear. Underlying pathophysiological mechanisms of PR prolongation leading to adverse cardiovascular events were poorly understood. We investigated the role of PR prolongation in pathophysiologically-related adverse cardiovascular events and underlying mechanisms.

**Methods:**

We prospectively investigated 597 high-risk cardiovascular outpatients (mean age 66 ± 11 yrs.; male 67%; coronary disease 55%, stroke 22%, diabetes 52%) for new-onset ischemic stroke, myocardial infarction (MI), congestive heart failure (CHF), and cardiovascular death. Vascular phenotype was determined by carotid intima-media thickness (IMT).

**Results:**

PR prolongation >200 ms was present in 79 patients (13%) at baseline. PR prolongation >200 ms was associated with significantly higher mean carotid IMT (1.05 ± 0.37 mm vs 0.94 ± 0.28 mm, *P = 0.010*). After mean study period of 63 ± 11 months, increased PR interval significantly predicted new-onset ischemic stroke (*P = 0.006*), CHF (*P = 0.040*), cardiovascular death (*P < 0.001*), and combined cardiovascular endpoints (*P < 0.001*) at cut-off >200 ms. Using multivariable Cox regression, PR prolongation >200 ms independently predicted new-onset ischemic stroke (HR 8.6, 95% CI: 1.9–37.8, *P = 0.005*), cardiovascular death (HR 14.1, 95% CI: 3.8–51.4, *P < 0.001*) and combined cardiovascular endpoints (HR 2.4, 95% CI: 1.30–4.43, *P = 0.005*). PR interval predicts new-onset MI at the exploratory cut-off >162 ms (C-statistic 0.70, *P = 0.001;* HR: 8.0, 95% CI: 1.65–38.85, *P = 0.010*).

**Conclusions:**

PR prolongation strongly predicts new-onset ischemic stroke, MI, cardiovascular death, and combined cardiovascular endpoint including CHF in coronary patients or risk equivalent. Adverse vascular function may implicate an intermediate pathophysiological phenotype or mediating mechanism.

**Electronic supplementary material:**

The online version of this article (doi:10.1186/s12872-017-0667-2) contains supplementary material, which is available to authorized users.

## Background

First-degree heart block, referred to as prolongation of PR interval beyond >200 ms on the electrocardiogram (ECG), has long been considered a benign functional variation [1]. Nevertheless, recent major studies including the ARIC [2] and Framingham Heart Study [3], re-ignited controversies over the pathological nature of PR prolongation by consistently demonstrating that PR prolongation was linked to risk of atrial fibrillation in the population. In the Framingham study, PR prolongation was further associated with increased risk of pacemaker implantation and all-cause death [3]. Nevertheless, the roles of PR prolongation in atherosclerotic cardiovascular (CV) disease, as well as outcome prediction in CV patients under clinical care, remained largely unknown. The Heart and Soul Study showed that PR prolongation predicted heart failure hospitalization and CV mortality in patients with stable coronary artery disease (CAD) [4]. We further showed that PR prolongation when incorporated into the CHADS2 and CHA2DS2-VASc scores, augments their predictive power for new-onset CV events [5]. Nevertheless, the individual role of PR prolongation, a precursor to atrial fibrillation, for new-onset ischemic events of stroke and myocardial infarction was not clearly established. Importantly, underlying pathophysiological mechanistic basis of how PR prolongation may lead to adverse CV outcomes remained also poorly understood.

In a prior study of 88 healthy subjects, PR prolongation was found independently correlated with endothelial dysfunction and increased pulse-wave velocity even in the absence of clinically manifest atherosclerotic disease [6], suggesting that adverse vascular function could be an intermediate pathological phenotype in subjects with PR prolongation progressing towards clinical events. As endothelial dysfunction indicates widespread functional adversity of the CV system as a collective, it will be of interest to dissect the role of PR prolongation in closely related CV pathological entities commonly characterized by abnormal vascular function, including myocardial infarction (MI) [7], ischemic stroke [8], and congestive heart failure (CHF) [9].

Therefore, we investigated in this prospective cohort study the role of PR prolongation as an independent predictor in a continuum of pathophysiologically-linked clinical events including ischemic stroke, MI, CHF, and CV death in high-risk CV patients, and the relationships between PR prolongation and indicator of abnormal vascular function.

## Methods

### Study population, design and clinical assessments

We studied 597 consecutive patients with CAD or risk equivalent recruited from outpatient medical specialty clinics, as prior described [5]. Among them, 328 patients had history of prior CAD (55%), 131 had ischemic stroke (22%), and 310 had diabetes mellitus (52%). All patients completed written informed consent. The study was adherent to the Declaration of Helsinki and was approved by the Ethics Committee, Hospital Authority (Hong Kong West)/University of Hong Kong.

During the study period Dec 2007 to May 2012, occurrence of CV death, new-onset ischemic stroke,

MI and CHF were retrieved from the central computerized clinical records archive of all public hospitals. Primary endpoints of the study were carotid IMT and clinical events of CV death, new-onset MI and ischemic stroke. Secondary Endpoints were new-onset CHF and combined CV endpoints, which included occurrences of any primary endpoints or CHF. Baseline demographic, clinical and laboratory assessments, clinical event definitions, and the inclusion/exclusion criteria were prior detailed elsewhere [5].

### Carotid Intima–Media Thickness (IMT)

As previously described in details [10], carotid IMT was assessed as the distance between lumen-intima and media-adventitia borders of the carotid arterial wall using electronic calipers under high-resolution ultrasound. Three measurements were taken at multiple sites on each side of the carotid arteries with resultant averaged estimate incorporating all measurements. Intraobserver variability analyses revealed satisfactory correlation (*R* = 0.97, *P < 0.001*).

### PR prolongation

Each participant had 12-lead ECG assessment after rested in the supine position for 5 min. PR interval prolongation was defined as >200 ms [1]. An exploratory cut-off of PR interval > 162 ms was further tested based on the median cut-off correlated with endothelial dysfunction in our earlier exploratory study of 88 healthy individuals [6]. Widened QRS duration was determined as >120 ms [11].

### Statistical analysis

Associations between PR interval and carotid IMT were examined by univariable and multivariable linear regression. Kaplan-Meier analysis was used to assess differences in survival as stratified by baseline PR interval status. A multivariable Cox proportional regression model was used to derive Hazard Ratios (HR) for incident CV events such that potential confounder variables with *p*-value ≤0.20 (based on univariable analysis) were entered. Findings were compared with aother Fully-Adjusted Model to verify robustness of study findings, such that variables a priori determined to be relevant were included (Additional file 1). The Vascular Function Model further dissects effect of carotid IMT adjustment in the estimates of PR interval as an independent predictor for CV events. SPSS Statistics (Version 20) was used for the above analyses.

## Results

### Baseline clinical characteristics

As shown in Table 1, PR prolongation >200 ms was present in 13% of patients (79/597). Patients with PR prolongation had higher mean age (*P < 0.001*) and greater male predominance (*P < 0.001*). There were no significant differences in history of diabetes mellitus, hyperlipidemia, smoking, physical activity, resting pulse rate, systolic/diastolic blood pressure, fasting glucose, HbA1c, and hsCRP (all *P* > 0.05). Lower LDL-cholesterol was noted in patients with PR prolongation (*P < 0.001*) with no differences in triglycerides and HDL-cholesterol (*P* > 0.05), which could be partially due to higher prevalence of statin use among patients with PR prolongation, albeit that was not statistically significant (*P* = 0.41). PR prolongation was associated with higher serum creatinine indicating impaired renal function (106.9 ± 59.6 versus 86.9 ± 29.2 mmol/L, *P = 0.005*), and a higher proportion of patients with PR prolongation had prior CAD (*P = 0.037*) or stroke (*P = 0.017*), with expectedly more prevalent use of aspirin (*P = 0.017*) and ACEI/ARB (*P = 0.008*). No between-groups difference of PR interval were found regarding the use of nodal-blocking agents including beta-blockers (use versus no use: 174.6 ± 27.1 ms versus 171.2 ± 28.5 ms, *P* = 0.15; linear regression (B = +3.3 [95% CI: -1.2 – 7.9], *P* = 0.39); and CCB (use versus no use: 173.7 ± 26.5 ms versus 172.2 ± 28.3 ms, *P* = 0.54; linear regression (B = +1.6 [95% CI: -3.5 – 6.6], *P* = 0.54). QRS duration was similar between groups (*P* = 0.14).

**Table 1:** Baseline characteristics of Participants by PR Interval Status (*n* = 597)^a^

	PR Interval ≤ 200 ms (*n* = 518)	PR Interval > 200 ms (*n* = 79)	*p*-value
Male [n (%)]	334 (65%)	67 (85%)	<0.001*
Age (years)	65.0 ± 11.1	70.5 ± 9.6	<0.001*
Body mass index (kgm^−2^)	25.2 ± 3.5	25.6 ± 3.2	0.34
Coronary Artery Disease [n (%)]	276 (53%)	52 (66%)	0.037*
Prior stroke [n (%)]	105 (21%)	26 (33%)	0.017*
Diabetes mellitus [n (%)]	274 (53%)	36 (46%)	0.23
Hypertension [n (%)]	330 (65%)	57 (72%)	0.20
Hyperlipidemia [n (%)]	320 (64%)	47 (60%)	0.48
Current/Past smoker [n (%)]	211 (42%)	43 (54%)	0.042
Regular Physical activity [n (%)]	185 (37%)	25 (12%)	0.40
Resting pulse rate (/min)	60.3 ± 21.2	63.7 ± 15.4	0.18
Systolic blood pressure (mmHg)	141.1 ± 20.5	140.2 ± 18.6	0.71
Diastolic blood pressure (mmHg)	79.0 ± 9.6	78.7 ± 9.3	0.83
LDL-cholesterol (mmol/L)	2.7 ± 0.7	2.4 ± 0.70	0.001*
HDL-cholesterol (mmol/L)	1.3 ± 0.3	1.2 ± 0.4	0.14
Tiglycerides (mmol/L)	1.5 ± 1.0	1.4 ± 0.7	0.37
Fasting glucose (mmol/L)	6.3 ± 2.1	6.1 ± 1.9	0.32
HbA1c (%)	7.0 ± 1.5	6.8 ± 1.2	0.22
hs-CRP (mg/L)	3.0 ± 8.9	2.7 ± 5.7	0.80
Serum creatinine (μmol/L)	86.9 ± 29.2	106.9 ± 59.6	0.005*
Medications:			
ACEI/ARB [n (%)]	268 (54%)	55 (70%)	0.008*
Beta-blockers [n (%)]	241 (49%)	43 (54%)	0.39
Calcium channel blockers [n (%)]	140 (28%)	22 (28%)	0.97
Aspirin [n (%)]	323 (65%)	62 (79%)	0.017*
Statin [n (%)]	285 (57%)	49 (62%)	0.41
PR interval (ms)	164.7 ± 19.0	222.6 ± 22.0	<0.001*
QRS duration (ms)	94.3 ± 27.1	99.0 ± 17.8	0.14
Mean Carotid IMT (mm)	0.94 ± 0.28	1.05 ± 0.37	0.010*

*HDL* high-density lipoprotein, *LDL* low-density lipoprotein, *HbA1c* glycosylated haemoglobin A1c, *hs-CRP* high-sensitivity C-reactive protein, *ACEI* angiotensin-converting enzyme inhibitors, *ARB* angiotensin receptor blockers, *IMT* intima-media thickness

*P-value < 0.05

^a^All values are $\overline{x}$ *±* SD, except where indicated otherwise

### PR prolongation and adverse vascular function

**Fig. 1:**
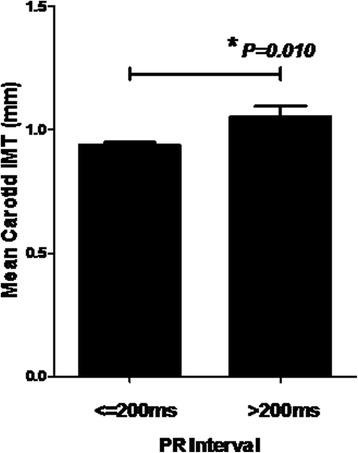
PR Prolongation and Mean Carotid Intima-Media Thickness (IMT). Patients with PR prolongation >200 ms had significantly higher mean carotid IMT (1.05 ± 0.37 versus 0.94 ± 0.28 mm, *P = 0.010*)

In this group of high-risk patients with CAD or risk equivalent, increased PR interval was positively associated with increased carotid IMT (Pearson *R* = 0.13, *P = 0.002*). Patients with PR prolongation had significantly higher mean carotid IMT (1.05 ± 0.37 versus 0.94 ± 0.28 mm, *P = 0.010,* Fig. 1). Adjusted for potential confounding variables including age, gender, prior stroke, smoking, body-mass index, resting pulse rate, systolic blood pressure, hs-CRP, fasting glucose, creatinine, and use of ACEI/ARB, CCB, beta-blockers, aspirin, and widened QRS interval, PR prolongation >200 ms remained independently associated with higher carotid IMT by +0.073 mm (95% CI: 0.003–0.143, *P = 0.041,* Additional file 1: Table S1). Repeated analysis by the Fully-Adjusted model produced similar results (B = +0.074 [95% CI: 0.002–0.147], *P = 0.044*). Other positive predictors for carotid IMT included age, prior stroke and systolic blood pressure. Statin use was independently associated with lower carotid IMT (*P = 0.041*). Diastolic blood pressure was no longer a significant predictor after adjusting for pulse pressure in relation to systolic blood pressure (*P* > 0.05). QRS duration was not associated with carotid IMT (*R* = 0.03, *P* = 0.55).

### Prediction estimate of PR prolongation for new-onset ischemic stroke and CV events

After mean study period of 63.4 ± 10.6 months, 21 (4%) patients developed new-onset ischemic stroke; 26 (4%) patients developed new-onset MI; 37 (6%) patients developed new-onset CHF; 27 (5%) patients had CV death and 87 (19%) patients developed any of these adverse CV events as a combined endpoint (Table 2a and b). Patients with PR prolongation >200 ms at baseline had significantly higher risk of new-onset ischemic stroke (*P = 0.006*), CHF (*P = 0.039*), CV death (*P < 0.001*), and combined CV endpoint (*P < 0.001*). PR interval length was not associated with risk of new-onset MI at cut-off >200 ms (*P* > 0.05) (primary cut-off) but a positive association was observed at the exploratory cut-off >162 ms (*P = 0.008*). ROC Curve analysis revealed overall significant prediction of PR interval for new-onset MI (C-statistic 0.70, *P = 0.001*).

**Table 2:** New-Onset Cardiovascular (CV) Events Stratified by Baseline PR Interval and QRS Duration

(a). PR interval						
New-Onset CV Events n (%)	Total (*n* = 597)	PR Interval < 200 ms (*n* = 518)	PR Interval > 200 ms (*n* = 79)	*P*-value	ROC C-Statistic	
					Estimate	*P-value*
Myocardial infarction	26 (4%)	20 (4%)	6 (8%)	0.130	0.70	0.001*
Ischemic stroke	21 (4%)	14 (3%)	7 (9%)	0.006*	0.50	0.99
Congestive heart failure	37 (6%)	28 (5%)	9 (11%)	0.040*	0.60	0.042*
CV death	27 (5%)	17 (3%)	10 (13%)	<0.001*	0.63	0.020*
Combined CV endpoints	87 (19%)	65 (13%)	22 (28%)	<0.001*	0.61	0.001*
(b). QRS duration						
New-Onset CV Events n (%)		QRS Duration <120 ms (*n* = 556)	QRS Duration >120 ms (*n* = 36)	*P*-value	ROC C-Statistic	
					Estimate	*P-value*
Myocardial infarction	-	22 (4%)	3 (8%)	0.21	0.55	0.37
Ischemic stroke	-	20 (4%)	1 (3%)	0.80	0.47	0.60
Congestive heart failure	-	30 (5%)	7 (19%)	0.001*	0.59	0.08
CV death	-	24 (4%)	3 (8%)	0.26	0.62	0.03*
Combined CV endpoints	-	76 (14%)	10 (28%)	0.020*	0.56	0.08

*P<0.05

#### CV death

**Fig. 2:**
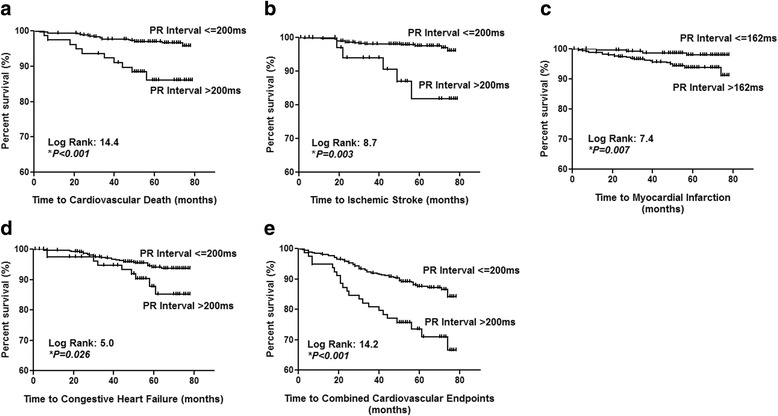
Kaplan-Meier Survival Curves for New-Onset Cardiovascular Events by PR Prolongation. Patients with PR prolongation >200 ms had significantly reduced survival from **a** cardiovascular death (log rank: 14.4, *P < 0.001*); **b** Ischemic Stroke (log rank: 8.7, *P = 0.003*); **d** CHF (log rank: 5.0, *P = 0.026*) **e** Combined Cardiovascular Endpoints (log rank: 14.2, *P < 0.001*). PR interval > 162 ms was associated with reduced survival from **c** new-onset MI (log rank: 7.4, *P = 0.007*)

Incidence rates for CV death in patients with PR interval ≤ 200 ms or >200 ms were respectively 0.6 and 1.8 per 100 person-years. Univariable analysis revealed that age, resting pulse rate, systolic blood pressure, serum hs-CRP, creatinine, widened QRS > 120 ms, and PR prolongation were all significant positive predictors for CV death (all *P < 0.05*). Furthermore, Kaplan-Meier analysis showed that subjects with PR prolongation >200 ms had significantly reduced survival (log rank: 14.4, *P < 0.001,* Fig. 2a). Multivariable Cox regression analysis showed that adjusted for potential confounders, PR prolongation >200 ms independently predicted substantially increased CV death (HR 6.53, 95% CI: 2.39–17.84, *P < 0.001*, Additional file 1: Table S2). Analyses by the Fully-Adjusted Model yielded similarly robust estimates (HR: 14.94, 95% CI: 3.99–55.92*, P < 0.001*). The Vascular Function Model showed that adjusting for carotid IMT did not materially alter the independent prediction by PR prolongation (HR: 16.40, 3.99–67.46, *P < 0.001*). Other independent positive predictors included HbA1c and hs-CRP (both *P < 0.05*). Higher BMI was associated with reduced CV death (*P < 0.05*). On the other hand, PR interval predicted total mortality with only marginal statistical significance (C-statistic 0.58, *P = 0.042*), and that it did not predict non-CV death (C-statistic 0.53, *P* = 0.63).

#### Ischemic stroke

Incidence rates for ischemic stroke in patients with PR interval ≤ 200 ms or >200 ms were respectively 0.5 and 1.9 per 100 person-years. Age, serum creatinine, carotid IMT, and PR prolongation >200 ms were all significant predicting variables for new-onset ischemic stroke (all *P < 0.05*). Furthermore, Kaplan-Meier analysis showed that PR prolongation >200 ms was significantly associated with reduced new-onset ischemic stroke-free survival (log rank: 8.7, *P = 0.003,* Fig. 2b). By multivariable Cox regression, PR prolongation >200 ms independently predicted increased risk of new-onset ischemic stroke (HR 3.76, 95% CI: 1.27–11.12, *P = 0.016*, Additional file 1: Table S3). Analysis using the Fully-Adjusted Model gave similarly robust estimates (HR: 5.36, 95% CI: 1.47–19.5*, P = 0.011*). Adjusting for carotid IMT in the Vascular Function Model, the independent predictive estimate by PR prolongation was only slightly attenuated (HR: 5.05, 95% CI: 1.34–19.10, *P = 0.017*).

### MI

Incidence rates for MI in patients with PR interval ≤ 162 ms or >162 ms were 0.3 and 1.3 per 100 person-years respectively. Age, prior CAD, resting pulse rate, systolic blood pressure, serum creatinine, history of ACEI/ARB use, carotid IMT, and PR prolongation >162 ms (exploratory cut-off) were all significant positive predictors for new-onset MI (all *P < 0.05*). However, the primary cut-off of PR interval > 200 ms was not associated with new-onset MI (*P* = 0.11). Kaplan-Meier analysis showed that patients with PR prolongation >162 ms had significantly reduced new-onset MI-free survival (log rank: 7.4, *P = 0.007,* Fig. 2c). Adjusting for potential confounders, PR prolongation >162 ms independently predicted increased risk of new-onset MI (HR: 8.0, 95% CI: 1.65–38.85, *P = 0.010*, Additional file 1: Table S4). Repeated analysis using the Vascular Function Model did not materially alter the independent prediction of new-onset MI by PR prolongation >162 m (HR: 8.05, 95% CI: 1.66–39.06, *P = 0.010*). Other independent positive predictors for MI included age and prior CAD. BMI was associated with reduced new-onset MI (*P < 0.05*).

### CHF

Incidence rates for CHF in patients with PR interval ≤ 200 ms or >200 ms were 1.1 and 2.4 per 100 person-years respectively. Age, resting pulse rate, systolic blood pressure, physical inactivity, prior CAD, history of aspirin/statin use, serum creatinine, hs-CRP, carotid IMT, QRS > 120 ms, and PR prolongation >200 ms were significant positive predictors of CHF (*all P < 0.05*). Patients with PR prolongation >200 ms had significantly reduced new-onset CHF-free survival (log rank: 5.0, *P = 0.026,* Fig. 2d). Nevertheless, after adjustment for potential confounders, PR prolongation >200 ms was no longer a significant predictor (HR: 1.29, 95% CI: 0.54–3.11, *P* = 0.56). Age, resting pulse rate, and history of aspirin use remained independent predictors of CHF (*all P < 0.05*).

### Combined CV endpoints

Incidence rates for combined CV endpoints in patients with PR interval ≤ 200 ms or >200 ms were 2.6 and 6.3 per 100 person-years respectively. Age, prior CAD, smoking, systolic blood pressure, resting pulse rate, hs-CRP, creatinine, history of ACEI/ARB use, QRS >120 ms, carotid IMT, and PR prolongation >200 ms were all significant positive predictors for increased combined CV endpoints (all *P < 0.05*, Additional file 1: Table S5). Kaplan-Meier analysis showed that patients with PR prolongation >200 ms had significantly reduced combined endpoint-free survival (log rank: 14.2, *P < 0.001,* Fig. 2e). Adjusted for potential confounders, PR prolongation >200 ms remained an independent predictor for increased combined CV endpoints (HR: 1.95, 95% CI: 1.11–3.43, *P = 0.020*). The Fully-Adjusted Model yielded similar estimates (HR: 2.40, 95% CI: 1.30–4.43*, P = 0.005*). Further adjustment for carotid IMT did not materially alter the independent prediction by PR prolongation (HR: 2.33, 95% CI: 1.26–4.32, *P = 0.007*). Other independent positive predictors included age, prior CAD, hs-CRP and creatinine (all *P < 0.05*).

### Clinical utility in the prediction of CV events

The clinical utility measures of sensitivity, specificity, positive predictive value (PPV) and negative predictive value (NPV) at specific cut-off points of PR interval in the prediction of each CV event were presented in Additional file 1: Table S6.

## Discussion

This is our first recognized study to show that PR prolongation alone is a strong and independent predictor for CV death, new-onset ischemic stroke and MI, and combined CV endpoints including CHF among patients with CAD or risk equivalent. We also assessed the vascular phenotype of these high-risk CV patients and found that PR prolongation was independently related to increased carotid IMT. Interestingly, despite the stated observations, adjustment for carotid IMT did not materially alter the prediction of PR prolongation for incident CV events, which suggests that atherosclerosis alone does not fully explain the pathophysiological mechanism along the pathway of PR prolongation leading to clinical events.

The clinical implications of this study are multi-fold. Firstly, as opposed to the traditional school of thought that considered PR prolongation as a benign ECG feature [1], this study robustly showed that PR prolongation is strongly predictive of a collective plethora of pathophysiologically related CV outcomes, including MI, ischemic stroke, CHF and CV death. Recent epidemiological studies (ARIC (2) and Framingham [3]) found higher risk of atrial fibrillation associated with PR prolongation. Our findings suggest that the pathophysiological implications of PR prolongation is far more than atrial fibrillation, and entails MI, ischemic stroke and CHF, all of which could be commonly characterized by impaired systemic vascular function [7–9]. PR prolongation alone, regardless of other pertinent CV risk factors [12] such as those delineated in the CHADS2 scores [5], is a simple and independent CV risk predictor. Secondly, the magnitude of excess risk of PR prolongation was substantial for various adverse outcomes consistently (new-onset ischemic stroke: 4-fold excess risk; new-onset MI, 7-fold excess risk; CV death: 15-fold excess risk), thus reflecting PR prolongation has strong utility for CV prediction among high-risk patients. Thirdly, while the recent Heart and Soul Study [4] showed that PR prolongation predicted heart failure hospitalization and CV death amongst patients with stable CAD, our study further showed that PR prolongation has strong predictive applicability for CV events in a much wider spectrum of high-risk patients with prior CAD or other risk equivalents, including diabetes and stroke. Fourthly, the escalated risk of new-onset MI emerged at a lower PR interval cut-off of >162 ms, consistent with our prior exploratory study in terms of subclinical changes in endothelial dysfunction [6], and calls into question that early pathological changes may well precede the conventional cut-off of PR prolongation at >200 ms.

Mechanisms underlying our clinical observation are likely multi-dimensional. Firstly, PR prolongation representing delayed atrioventricular conduction could be an ECG manifestation of CV ageing [13]. Atherosclerosis is increasingly recognized as a multi-factorial degenerative disease that is closely related to the aging process. Our analysis indicated that PR prolongation could be an excellent clinical marker of CV ageing since adjustment for chronological age did not obliterate the prediction of carotid IMT and CV events by PR prolongation. Secondly, myocardial fibrosis may involve the cardiac conduction system and is closely linked to the presence of CV risk factors including CAD [14], hypertension [15], diabetes [16], hyperlipidemia [17] and inflammation [18]. Thus PR prolongation may be a risk marker that reflects the clustering of these important risk factors. Nevertheless, PR prolongation may also reflect higher underlying vagal tone [19], especially in the young [1], other than degenerative damage of the cardiac conduction system which predominantly affects the elderly [13]. In our study, comprehensive adjustment for important CV risk factors, even when including widened QRS complex signifying overt ventricular electrical dyssynchrony or widespread conduction system disease, did not abolish PR prolongation as an independent CV predictor. Therefore a third potential explanation arises: that PR prolongation may be causal to adverse CV events. Indeed, PR prolongation, be it vagal- or degenerative-predominant, may mediate its adverse vascular effects via increased intra-atrial pressure consequential to slowed atrioventricular conduction, resulting in neurohormonal activation [20]. Adverse neurohormonal changes including raised aldosterone may be reversible on sinus rhythm reversion [21]. Aldosterone is associated with a constellation of pro-atherosclerotic changes including raised inflammatory response [22], reduced circulating endothelial progenitor cell quantity and function [23] resulting in increased arterial stiffness [24]. Importantly, mineralocorticoid receptor blockade has been shown clinically to improve atherosclerotic changes [25]. Indeed, if neurohormonal activation is the explanatory mechanism that underlies PR prolongation-related vascular dysfunction, such findings may translate into potentially important preventive implications through pharmacological modulation of the neurohormonal system.

Vascular function as indicated by carotid IMT is a strong, independent and consistent predictor of both incident ischemic stroke and CV events overall among healthy subjects from diverse ethnicities [26]. Increased carotid IMT also predicts elevated risks of CV complications in patients with diabetes [27], hypertension [28] and prior atherosclerotic diseases [29]. In this study, the magnitude of increased carotid IMT by +0.07 mm accounted for by PR prolongation is clinically significant, since each 0.1 mm increase of carotid IMT could translate into 18% excess risk of recurrent CV events [30]. On top of their greater susceptibility to CV events in general, patients with prior CAD also have increased risk of incident ischemic stroke, with diabetes being an additional independent predictor [31, 32]. Our study further suggests that the adverse prognostic value of PR prolongation is independent and over that of carotid IMT for CV events in high-risk patients. Whether impaired vascular function is indeed along the pathophysiological pathway or simply a bystander will require further mechanistic studies. Future studies could be strengthened by incorporating multiple vascular assessment modalities to ensure consistent findings and scrutinise further mechanisitc insights.

It should be noted that from our study, the magnitude of risk associations of PR interval prolongation with new-onset CV events was striking, and that IMT did not fully explain such associations, suggesting that alternative mediating pathophysiological mechanisms could be present. Also, the HR estimates are relatively large. Despite our meticulous efforts in the study design and data analysis to carefully minimize biases or residual confounding, potential risk of bias and confounding cannot be excluded totally. Findings from the exploratory endpoint analyses should also best be verified in another independent study. Furthermore, the study of incident atrial fibrillation was not included in the current study, which should be explored in the future.

## Conclusions

In this prospective study of patients with CAD or risk-equivalent, PR prolongation strongly and independently predicts CV death, new-onset MI, ischemic stroke, and combined CV endpoints including CHF. Increased risk of MI emerged at a cut-off as low as PR interval > 162 ms. Whether adverse vascular function in PR prolongation is an intermediate phenotype or represent a mediating pathway prior to clinical events warrants further investigations.

## Additional file

Additional file 1:
**Table S1.** Univariable and Multivariable Predictors for Carotid IMT. **Table S2.** Univariable and Multivariable Predictors for Cardiovascular Death. **Table S3.** Univariable and Multivariable Predictors for New-Onset Ischemic Stroke. **Table S4.** Univariable and Multivariable Predictors for New-Onset Myocardial Infarction. **Table S5.** Univariable and Multivariable Predictors for Combined Cardiovascular Endpoints of New-Onset Myocardial Infarction, Ischemic Stroke, Congestive Heart Failure and Cardiovascular Death. **Table S6.** Estimates of Sensitivity (Se), Specificity (Sp), Positive Predictive Value (PPV) and Negative Predictive Value (NPV) of PR Interval in the Prediction for Cardiovascular Events. (DOC 444 kb)

## Abbreviations

ACEI: Angiotensin-Converting Enzyme Inhibitors
BMI: Body-Mass Index
CAD: Coronary Artery Disease
CCB: Calcium Channel Blockers
CHF: Congestive Heart Failure
hbA1c: Glycosylated Hemoglobin A1c
HDL/LDL: High/Low-Density Lipoprotein
hs-CRP: High-Sensitivity C-Reactive Protein
IMT: Intima-Media Thickness
MI: Myocardial Infarction
