# Supplementary material for: PR interval prolongation in coronary patients or risk equivalent: excess risk of ischemic stroke and vascular pathophysiological insights
Source: BMC Cardiovasc Disord. 2017 Aug 24;17:233. doi: 10.1186/s12872-017-0667-2 (PMC5571504; doi:10.1186/s12872-017-0667-2)
Supplement: Additional file 1: — Table S1. Univariable and Multivariable Predictors for Carotid IMT. Table S2. Univariable and Multivariable Predictors for Cardiovascular Death. Table S3. Univariable and Multivariable Predictors for New-Onset Ischemic Stroke. Table S4. Univariable and Multivariable Predictors for New-Onset Myocardial Infarction. Table S5. Univariable and Multivariable Predictors for Combined Cardiovascular Endpoints of New-Onset Myocardial Infarction, Ischemic Stroke, Congestive Heart Failure and Cardiovascular Death. Table S6. Estimates of Sensitivity (Se), Specificity (Sp), Positive Predictive Value (PPV) and Negative Predictive Value (NPV) of PR Interval in the Prediction for Cardiovascular Events. (DOC 444 kb) [file 12872_2017_667_MOESM1_ESM.doc]

**ADDITIONAL FILE 1: TABLE S1. Univariable and Multivariable Predictors for Carotid Intima-Media Thickness (IMT) ^1^**.

|  | **Univariable Model** | | **Multivariable Model** ^3^ | | **Fully-Adjusted Model** ^4^ | |
| --- | --- | --- | --- | --- | --- | --- |
|  | B [95%CI] *^2^* | P-value | B [95%CI] | P-value | B [95%CI] | P-value |
| **Age (years)** | 0.008 [0.006 to 0.010] | *<0.001** | 0.006 [0.003 to 0.009] | *<0.001** | 0.005 [0.002 to 0.008] | *0.001** |
| **Male** | 0.090 [0.040 to 0.141] | *<0.001** | 0.010 [-0.058 to 0.078] | 0.77 | 0.027 [-0.048 to 0.102] | 0.48 |
| **Body mass index (kgm^-2^)** | -0.008 [-0.015 to -0.001] | *0.021** | -0.007 [-0.015 to 0.001] | 0.069 | -0.007 [-0.015 to 0.001] | 0.097 |
| **Coronary Artery Disease** | 0.010 [-0.038 to 0.058] | 0.69 | - |  | 0.057 [-0.033 to 0.146] | 0.22 |
| **Prior stroke** | 0.135 [-0.078 to 0.192] | *<0.001** | 0.112 [0.048 to 0.175] | *0.001** | 0.106 [0.023 to 0.190] | *0.013** |
| **Diabetes mellitus** | 0.005 [-0.042 to 0.053] | 0.82 | - |  | 0.053 [-0.019 to 0.126] | 0.15 |
| **Current/Past smoker** | 0.119 [0.071 to 0.166] | *<0.001** | 0.050 [-0.006 to 0.105] | 0.081 | 0.044 [-0.013 to 0.102] | 0.13 |
| **Regular Physical activity** | 0.020 [-0.030 to 0.070] | 0.43 | - |  | 0.014 [-0.044 to 0.072] | 0.63 |
| **Resting pulse rate (bpm)** | 0.002 [0.001 to 0.004] | *<0.001** | 0.001 [0.000 to 0.003] | 0.11 | 0.001 [-0.001 to 0.003] | 0.25 |
| **Systolic BP (mmHg)** | 0.003 [0.002 to 0.004] | *<0.001** | 0.001 [0.000 to 0.003] | 0.073 | 0.003 [0.001 to 0.005] | *0.007** |
| **Diastolic BP (mmHg)** | -0.001 [-0.004 to 0.001] | 0.31 | - |  | -0.004 [-0.008 to 0.000] | *0.031** |
| **LDL-cholesterol (mmol/L)** | -0.013 [-0.045 to 0.020] | 0.44 | - |  | 0.016 [-0.023 to 0.056] | 0.41 |
| **HDL-cholesterol (mmol/L)** | -0.070 [-0.139 to 0.000] | 0.051 | - |  | -0.055 [-0.141 to 0.031] | 0.21 |
| **Triglycerides (mmol/L)** | -0.009 [-0.033 to 0.015] | 0.47 | - |  | -0.001 [-0.027 to 0.025] | 0.96 |
| **Fasting glucose (mmol/L)** | -0.014 [-0.026 to -0.002] | *0.024** | -0.003 [-0.015 to 0.009] | 0.57 | -0.015 [-0.035 to 0.005] | 0.14 |
| **HbA1c (%)** | -0.004 [-0.021 to 0.013] | 0.63 | - |  | 0.007 [-0.023 to 0.038] | 0.64 |
| **hs-CRP (mg/L)** | 0.003 [0.000 to 0.006] | *0.024** | 0.002 [-0.001 to 0.005] | 0.18 | 0.002 [-0.001 to 0.005] | 0.11 |
| **Serum creatinine (µmol/L)** | 0.001 [0.001 to 0.002] | *<0.001** | 0.000 [-0.001 to 0.001] | 0.64 | 0.000 [-0.001 to 0.001] | 0.66 |
| **Medications:** |  |  |  |  |  |  |
| ACEI/ ARB | 0.098 [0.050 to 0.147] | *<0.001** | 0.059 [0.007 to 0.110] | *0.025** | 0.054 [0.000 to 0.108] | 0.051 |
| Beta-blockers | 0.033 [-0.016 to 0.083] | 0.185 | 0.001 [-0.053 to 0.056] | 0.96 | 0.003 [-0.054 to 0.061] | 0.91 |
| Calcium channel blockers | 0.066 [0.012 to 0.120] | *0.017** | 0.053 [-0.006 to 0.111] | 0.078 | 0.036 [-0.025 to 0.096] | 0.25 |
| Aspirin | 0.092 [0.040 to 0.143] | *<0.001** | 0.001 [-0.063 to 0.065] | 0.98 | 0.035 [-0.043 to 0.114] | 0.38 |
| Statin | -0.007 [-0.056 to 0.043] | 0.79 | - |  | -0.074 [-0.145 to -0.003] | *0.041** |
| **Widened QRS >120ms** | 0.091 [-0.009 to 0.190] | 0.074 | -0.031 [-0.132 to 0.070] | 0.55 | -0.033 [-0.135 to 0.069] | 0.53 |
| **PR prolongation >200ms** | 0.116 [0.047 to 0.186] | *0.001** | 0.073 [0.003 to 0.143] | *0.041** | 0.074 [0.002 to 0.147] | *0.044** |

*^1^* Absolute change estimates and 95% confidence interval of carotid IMT (mm) explained by variable of interest as estimated by univariable and multivariable linear regression;

*^2^* 95% confidence interval in parentheses;

^3^Adjusted for potential confounders as defined from univariable analysis with p-value <0.20;

^4^ Adjusted for all potentially confounding variables as defined a priori.

Abbreviations: BP, blood pressure; HDL, high-density lipoprotein; LDL, low-density lipoprotein; HbA1c, glycosylated haemoglobin A1c;

hs-CRP, high-sensitivity C-reactive protein; ACEI, angiotensin-converting enzyme inhibitors; ARB, angiotensin receptor blockers; IMT,

intima-media thickness

**P<0.05*

**ADDITIONAL FILE 1 TABLE S2.** Univariable and Multivariable Predictors for Cardiovascular Death.

|  | **Univariable Model** | | **Multivariable Model** ^2^ | | **Fully-Adjusted Model** ^3^ | | **Vascular Function Model** ^4^ | |
| --- | --- | --- | --- | --- | --- | --- | --- | --- |
|  | HR [95%CI] *^1^* | P-value | HR [95%CI] *^1^* | P-value | HR [95%CI] *^1^* | P-value | HR [95%CI] *^1^* | P-value |
| **Male** | 2.24 [0.85 to 5.92] | 0.10 | 0.61 [0.17 to 2.24] | 0.45 | 0.59 [0.13 to 2.72] | 0.59 | 0.95 [0.18 to 5.08] | 0.95 |
| **Age (years)** | 1.09 [1.04 to 1.14] | *<0.001** | 1.05 [0.99 to 1.12] | 0.11 | 1.04 [0.96 to 1.13] | 0.33 | 1.03 [0.95 to 1.12] | 0.48 |
| **Body mass index (kgm^-2^)** | 0.92 [0.82 to 1.03] | 0.15 | 0.80 [0.68 to 0.95] | *0.010** | 0.76 [0.61 to 0.94] | *0.010** | 0.78 [0.62 to 0.97] | *0.025** |
| **Coronary Artery Disease** | 1.30 [0.60 to 2.82] | 0.51 |  |  | 0.56 [0.08 to 3.98] | 0.56 | 0.66 [0.10 to 4.39] | 0.66 |
| **Prior stroke** | 1.61 [0.72 to 3.60] | 0.25 |  |  | 0.34 [0.05 to 2.49] | 0.29 | 0.40 [0.05 to 3.06] | 0.38 |
| **Diabetes mellitus** | 1.60 [0.73 to 3.50] | 0.24 |  |  | 0.47 [0.10 to 2.20] | 0.34 | 0.30 [0.06 to 1.59] | 0.16 |
| **Current/Past smoker** | 1.95 [0.91 to 4.21] | 0.087 | 1.04 [0.72 to 5.28] | 0.19 | 1.35 [0.43 to 4.31] | 0.61 | 1.40 [0.40 to 4.88] | 0.60 |
| **Regular Physical activity** | 0.80 [0.36 to 1.79] | 0.58 |  |  | 0.59 [0.17 to 2.10] | 0.42 | 0.75 [0.22 to 2.56] | 0.64 |
| **Resting pulse rate (bpm)** | 1.02 [1.00 to 1.04] | *0.019** | 1.02 [0.98 to 1.05] | 0.35 | 1.02 [0.98 to 1.07] | 0.28 | 1.02 [0.98 to 1.07] | 0.34 |
| **Systolic BP (mmHg)** | 1.03 [1.01 to 1.05] | *<0.001** | 1.03 [1.00 to 1.05] | *0.040** | 1.02 [0.98 to 1.07] | 0.32 | 1.03 [0.98 to 1.08] | 0.25 |
| **Diastolic BP (mmHg)** | 1.02 [0.98 to 1.06] | 0.29 |  |  | 1.03 [0.95 to 1.11] | 0.53 | 1.01 [0.93 to 1.10] | 0.81 |
| **LDL-cholesterol(mmol/L)** | 0.63 [0.35 to 1.13] | 0.63 |  |  | 0.62 [0.21 to 1.87] | 0.39 | 0.53 [0.17 to 1.71] | 0.29 |
| **HDL-cholesterol(mmol/L)** | 0.87 [0.26 to 2.85] | 0.81 |  |  | 1.78 [0.3 to 10.84] | 0.53 | 1.75 [0.24 to 12.9] | 0.58 |
| **Triglycerides (mmol/L)** | 0.73 [0.40 to 1.32] | 0.29 |  |  | 0.63 [0.26 to 1.56] | 0.32 | 0.54 [0.18 to 1.62] | 0.27 |
| **Fasting glucose (mmol/L)** | 0.94 [0.75 to 1.17] | 0.58 |  |  | 0.71 [0.47 to 1.09] | 0.12 | 0.76 [0.49 to 1.19] | 0.24 |
| **HbA1c (%)** | 1.14 [0.88 to 1.46] | 0.32 |  |  | 2.19 [1.29 to 3.72] | *0.004** | 2.29 [1.28 to 4.08] | *0.005** |
| **hs-CRP (mg/L)** | 1.06 [1.02 to 1.09] | *0.001** | 1.06 [1.03 to 1.09] | *<0.001** | 1.09 [1.03 to 1.15] | *0.004** | 1.13 [1.02 to 1.25] | *0.025** |
| **Serum creatinine (µmol/L)** | 1.01 [1.009 to 1.018] | *<0.001** | 1.01 [0.998 to 1.02] | 0.15 | 1.01 [1.00 to 1.02] | 0.072 | 1.01 [0.99 to 1.02] | 0.41 |
| **Medications:** |  |  |  |  |  |  |  |  |
| ACEI/ ARB | 1.93 [0.85 to 4.42] | 0.12 | 1.09 [0.40 to 2.93] | 0.87 | 1.08 [0.33 to 3.52] | 0.90 | 0.88 [0.25 to 3.08] | 0.85 |
| Beta-blockers | 1.33 [0.62 to 2.85] | 0.47 |  |  | 0.66 [0.21 to 2.04] | 0.47 | 0.73 [0.23 to 2.37] | 0.60 |
| Calcium channel blockers | 1.51 [0.69 to 3.30] | 0.30 |  |  | 2.10 [0.56 to 7.86] | 0.27 | 2.06 [0.51 to 8.35] | 0.31 |
| Aspirin | 2.28 [0.86 to 6.05] | 0.097 | 2.62 [0.59 to 11.6] | 0.20 | 8.09 [0.9 to 69.5] | 0.057 | 16.2 [0.8 to 320.1] | 0.067 |
| Statin | 0.85 [0.40 to 1.81] | 0.67 |  |  | 0.28 [0.06 to 1.34] | 0.11 | 0.37 [0.07 to 1.89] | 0.23 |
| **Widened QRS >120ms** | 1.99 [0.60 to 6.62] | 0.26 |  |  | 2.01 [0.4 to 10.3] | 0.40 | 1.60 [0.29 to 8.79] | 0.59 |
| **Carotid IMT (mm)** | 6.05 [2.98 to 12.27] | *<0.001** |  |  |  |  | 1.80 [0.40 to 8.09] | 0.44 |
| **PR prolongation >200ms** | 4.05 [1.85 to 8.84] | *<0.001** | 6.53 [2.39 to 17.84] | *<0.001** | 14.94  [3.99 to 55.92] | *<0.001** | 16.40  [3.99 to 67.46] | *<0.001** |

*^1^* Hazard ratio (HR) estimates and 95% confidence interval of cardiovascular death predicted by variable of interest as derived from univariable and multivariable Cox regression;

^2^Adjusted for potential confounders as defined as from univariable analysis with p-value <0.20;

^3^ Adjusted for all potentially confounding variables as defined *a priori*.

^4^ Adjusted for all potentially confounding variables as defined *a priori* and vascular function (carotid IMT)

Abbreviations as **Supp Table 1**.

**P<0.05*

**SADDITIONAL FILE 1 TABLE S3.** Univariable and Multivariable Predictors for New-Onset Ischemic Stroke.

|  | **Univariable Model** | | **Multivariable Model** ^2^ | | **Fully-Adjusted Model** ^3^ | | **Vascular Function Model** ^4^ | |
| --- | --- | --- | --- | --- | --- | --- | --- | --- |
|  | HR [95%CI] *^1^* | P-value | HR [95%CI] *^1^* | P-value | HR [95%CI] *^1^* | P-value | HR [95%CI] *^1^* | P-value |
| **Male** | 1.65 [0.61 to 4.52] | 0.33 |  |  | 0.68 [1.11 to 4.34] | 0.68 | 0.52 [0.08 to 3.37] | 0.49 |
| **Age (years)** | 1.07 [1.01 to 1.12] | *0.012** | 1.06 [0.99 to 1.13] | 0.089 | 1.05 [0.96 to 1.15] | 0.25 | 1.04 [0.95 to 1.14] | 0.41 |
| **Body mass index (kgm^-2^)** | 0.92 [0.80 to 1.05] | 0.22 |  |  | 0.95 [0.79 to 1.13] | 0.55 | 0.96 [0.80 to 1.15] | 0.65 |
| **Coronary Artery Disease** | 0.47 [0.19 to 1.16] | 0.10 | 1.20 [0.27 to 5.37] | 0.81 | 1.38 [0.20 to 9.44] | 0.74 | 1.22 [0.18 to 8.34] | 0.84 |
| **Prior stroke** | 2.22 [0.89 to 5.59] | 0.089 | 2.23 [0.66 to 7.59] | 0.20 | 2.61 [0.33 to 20.9] | 0.37 | 2.34 [0.28 to 19.8] | 0.44 |
| **Diabetes mellitus** | 1.86 [0.75 to 4.62] | 0.18 | 1.56 [0.49 to 4.99] | 0.45 | 2.54 [0.47 to 13.9] | 0.28 | 2.40 [0.43 to 13.3] | 0.32 |
| **Current/Past smoker** | 2.05 [0.84 to 5.02] | 0.12 | 1.50 [0.49 to 4.62] | 0.48 | 2.04 [0.53 to 7.82] | 0.30 | 1.77 [0.43 to 7.35] | 0.43 |
| **Regular Physical activity** | 1.53 [0.63 to 3.71] | 0.35 |  |  | 1.68 [0.48 to 5.90] | 0.42 | 1.73 [0.48 to 6.18] | 0.40 |
| **Resting pulse rate (bpm)** | 1.00 [0.98 to 1.02] | 0.99 |  |  | 0.98 [0.93 to 1.02] | 0.29 | 0.98 [0.93 to 1.02] | 0.26 |
| **Systolic BP (mmHg)** | 1.02 [0.998 to 1.037] | 0.079 | 1.00 [0.98 to 1.03] | 0.85 | 1.01 [0.95 to 1.06] | 0.84 | 0.99 [0.94 to 1.05] | 0.79 |
| **Diastolic BP (mmHg)** | 1.01 [0.96 to 1.05] | 0.79 |  |  | 1.03 [0.94 to 1.12] | 0.57 | 1.05 [0.95 to 1.15] | 0.34 |
| **LDL-cholesterol(mmol/L)** | 1.14 [0.62 to 2.13] | 0.67 |  |  | 0.97 [0.37 to 2.55] | 0.95 | 0.91 [0.32 to 2.56] | 0.86 |
| **HDL-cholesterol(mmol/L)** | 2.08 [0.68 to 6.37] | 0.20 | 1.80 [0.46 to 6.99] | 0.40 | 2.64 [0.43 to 16.4] | 0.30 | 2.78 [0.42 to 18.33] | 0.29 |
| **Triglycerides (mmol/L)** | 0.52 [0.12 to 1.25] | 0.14 | 0.58 [0.20 to 1.69] | 0.31 | 0.68 [0.22 to 2.06] | 0.49 | 0.58 [0.17 to 1.97] | 0.38 |
| **HbA1c (%)** | 0.94 [0.67 to 1.34] | 0.74 |  |  | 0.70 [0.36 to 1.38] | 0.31 | 0.75 [0.38 to 1.46] | 0.39 |
| **hs-CRP (mg/L)** | 0.99 [0.88 to 1.12] | 0.89 |  |  | 1.00 [0.87 to 1.14] | 0.94 | 0.98 [0.85 to 1.12] | 0.74 |
| **Serum creatinine (µmol/L)** | 1.01 [1.00 to 1.02] | *0.015** | 1.01 [0.10 to 1.02] | 0.16 | 1.01 [0.99 to 1.03] | 0.22 | 1.01 [0.998 to 1.03] | 0.089 |
| **Medications:** |  |  |  |  |  |  |  |  |
| ACEI/ ARB | 1.03 [0.41 to 2.63] | 0.94 |  |  | 1.30 [0.39 to 4.32] | 0.67 | 1.31 [0.37 to 4.65] | 0.67 |
| Beta-blockers | 1.15 [0.45 to 2.93] | 0.77 |  |  | 1.70 [0.47 to 6.20] | 0.42 | 1.52 [0.40 to 5.77] | 0.54 |
| Calcium channel blockers | 1.66 [0.64 to 4.29] | 0.30 |  |  | 1.54 [0.41 to 5.86] | 0.52 | 1.44 [0.37 to 5.56] | 0.60 |
| Aspirin | 0.84 [0.32 to 2.20] | 0.73 |  |  | 1.80 [0.30 to 12.1] | 0.55 | 1.65 [0.22 to 12.21] | 0.62 |
| Statin | 0.41 [0.15 to 1.10] | 0.077 | 0.37 [0.08 to 1.66] | 0.20 | 0.26 [0.05 to 1.45] | 0.12 | 0.28 [0.05 to 1.74] | 0.17 |
| **Widened QRS >120ms** | 0.80 [0.11 to 5.96] | 0.83 |  |  | 1.13 [0.12 to 10.8] | 0.91 | 1.19 [0.11 to 12.48] | 0.88 |
| **Carotid IMT (mm)** | 5.95 [2.67 to 13.26] | *<0.001** |  |  |  |  | 4.77 [0.86 to 26.37] | 0.073 |
| **PR prolongation >200ms** | 3.58 [1.45 to 8.88] | *0.006** | 3.76 [1.27 to 11.12] | *0.016** | 5.36 [1.47 to 19.5] | *0.011** | 5.05 [1.34 to 19.10] | *0.017** |

*^1^* Hazard ratio (HR) estimates and 95% confidence interval of new-onset ischemic stroke predicted by variable of interest as derived from univariable and multivariable Cox regression;

^2^Adjusted for potential confounders as defined as from univariable analysis with p-value <0.20;

^3^ Adjusted for all potentially confounding variables as defined *a priori*.

^4^ Adjusted for all potentially confounding variables as defined *a priori* and vascular function (carotid IMT)

Abbreviations as **Supp Table 1**.

**P<0.05*

**ADDITIONAL FILE 1 TABLE S4.** Univariable and Multivariable Predictors for New-Onset Myocardial Infarction.

|  | **Univariable Model** | | **Multivariable Model** ^2^ | | **Fully-Adjusted Model** ^3^ | | **Vascular Function Model** ^4^ | |
| --- | --- | --- | --- | --- | --- | --- | --- | --- |
|  | HR [95%CI] *^1^* | P-value | HR [95%CI] *^1^* | P-value | HR [95%CI] *^1^* | P-value | HR [95%CI] *^1^* | P-value |
| **Male** | 0.96 [0.43 to 2.15] | 0.91 |  |  | 0.35 [0.08 to 1.58] | 0.17 | 0.31 [0.07 to 1.45] | 0.14 |
| **Age (years)** | 1.14 [1.08 to 1.20] | *<0.001** | 1.11 [1.05 to 1.18] | <0.001* | 1.11 [1.02 to 1.19] | *0.010** | 1.11 [1.02 to 1.19] | *0.011** |
| **Body mass index (kgm^-2^)** | 0.97 [0.87 to 1.09] | 0.59 |  |  | 0.82 [0.67 to 0.99] | *0.038** | 0.82 [0.67 to 0.99] | *0.042** |
| **Coronary Artery Disease** | 4.04 [1.50 to 10.84] | *0.006** | 3.03 [0.93 to 9.81] | 0.065 | 32.44 [4.9 to 214.6] | *<0.001** | 28.91 [4.4 to 189.7] | *<0.001** |
| **Prior stroke** | 0.79 [0.29 to 2.13] | 0.64 |  |  | 2.02 [0.53 to 7.69] | 0.30 | 1.91 [0.50 to 7.24] | 0.34 |
| **Diabetes mellitus** | 1.56 [0.70 to 3.46] | 0.28 |  |  | 3.04 [0.72 to 12.74] | 0.13 | 3.11 [0.73 to 13.22] | 0.13 |
| **Current/Past smoker** | 1.62 [0.72 to 3.61] | 0.24 |  |  | 2.14 [0.58 to 7.90] | 0.26 | 2.04 [0.54 to 7.68] | 0.29 |
| **Regular Physical activity** | 0.92 [0.40 to 2.11] | 0.83 |  |  | 0.89 [0.25 to 3.14] | 0.86 | 0.85 [0.24 to 2.99] | 0.81 |
| **Resting pulse rate (bpm)** | 1.02 [1.00 to 1.04] | *0.035** | 1.01 [0.97 to 1.04] | 0.72 | 1.00 [0.96 to 1.04] | 0.93 | 1.00 [0.95 to 1.04] | 0.93 |
| **Systolic BP (mmHg)** | 1.03 [1.01 to 1.04] | *0.002** | 1.00 [0.98 to 1.03] | 0.88 | 1.04 [0.99 to 1.09] | 0.13 | 1.03 [0.99 to 1.08] | 0.17 |
| **Diastolic BP (mmHg)** | 0.98 [0.94 to 1.03] | 0.43 |  |  | 0.93 [0.85 to 1.02] | 0.13 | 0.94 [0.86 to 1.03] | 0.20 |
| **LDL-cholesterol(mmol/L)** | 1.06 [0.64 to 1.76] | 0.83 |  |  | 2.26 [0.89 to 5.76] | 0.087 | 2.35 [0.92 to 5.99] | 0.074 |
| **HDL-cholesterol(mmol/L)** | 0.75 [0.23 to 2.43] | 0.64 |  |  | 0.63 [0.11 to 3.65] | 0.61 | 0.71 [0.12 to 4.13] | 0.70 |
| **Triglycerides (mmol/L)** | 0.96 [0.64 to 1.46] | 0.86 |  |  | 0.68 [0.35 to 1.32] | 0.26 | 0.68 [0.34 to 1.33] | 0.26 |
| **Fasting glucose (mmol/L)** | 1.04 [0.87 to 1.26] | 0.66 |  |  | 1.12 [0.78 to 1.60] | 0.53 | 1.10 [0.77 to 1.58] | 0.59 |
| **HbA1c (%)** | 1.20 [0.96 to 1.51] | 0.11 | 1.25 [0.99 to 1.59] | 0.062 | 1.02 [0.61 to 1.70] | 0.94 | 1.02 [0.61 to 1.70] | 0.95 |
| **hs-CRP (mg/L)** | 1.00 [0.91 to 1.09] | 0.90 |  |  | 0.97 [0.85 to 1.12] | 0.70 | 0.97 [0.85 to 1.11] | 0.67 |
| **Serum creatinine (µmol/L)** | 1.01 [1.007 to 1.018] | *<0.001** | 1.00 [0.996 to 1.01] | 0.29 | 1.01 [1.00 to 1.02] | 0.30 | 1.01 [1.00 to 1.02] | 0.21 |
| **Medications:** |  |  |  |  |  |  |  |  |
| ACEI/ ARB | 2.64 [1.06 to 6.63] | *0.038** | 2.17 [0.81 to 5.82] | 0.13 | 3.09 [0.92 to 10.41] | 0.068 | 3.41 [0.96 to 12.16] | 0.058 |
| Beta-blockers | 1.19 [0.54 to 2.62] | 0.66 |  |  | 0.44 [0.14 to 1.43] | 0.17 | 0.44 [0.13 to 1.43] | 0.17 |
| Calcium channel blockers | 2.08 [0.94 to 4.58] | 0.070 | 2.37 [0.98 to 5.71] | 0.055 | 2.89 [0.90 to 9.31] | 0.075 | 2.90 [0.90 to 9.38] | 0.075 |
| Aspirin | 0.90 [0.40 to 2.06] | 0.81 |  |  | 0.34 [0.10 to 1.16] | 0.084 | 0.36 [0.11 to 1.20] | 0.096 |
| Statin | 1.44 [0.63 to 3.28] | 0.39 |  |  | 0.67 [0.18 to 2.53] | 0.55 | 0.63 [0.16 to 2.40] | 0.50 |
| **Widened QRS >120ms** | 2.24 [0.64 to 7.14] | 0.22 |  |  | 0.65 [0.12 to 3.57] | 0.62 | 0.66 [0.12 to 3.67] | 0.64 |
| **Carotid IMT (mm)** | 4.36 [1.95 to 9.77] | *<0.001** |  |  |  |  | 1.14 [0.26 to 4.99] | 0.86 |
| **PR Prolongation** |  |  |  |  |  |  |  |  |
| **>200ms (primary cut-off)** | 2.10 [0.84 to 5.23] | 0.11 | 1.09 [0.41 to 2.93] | 0.87 | 2.10 [0.62 to 7.08] | 0.24 | 2.00 [0.58 to 6.88] | 0.27 |
| **>162ms (exploratory)** | 3.93 [1.35 to 11.41] | *0.012** | 2.57 [0.86 to 7.65] | 0.091 | 8.0 [1.65 to 38.85] | *0.010** | 8.05 [1.66 to 39.06] | *0.010** |

*^1^* Hazard ratio (HR) estimates and 95% confidence interval of new-onset myocardial infarction predicted by variable of interest as derived from univariable and multivariable Cox regression;

^2^Adjusted for potential confounders as defined as from univariable analysis with p-value <0.20;

^3^ Adjusted for all potentially confounding variables as defined *a priori*.

^4^ Adjusted for all potentially confounding variables as defined *a priori* and vascular function (carotid IMT)

Abbreviations as **Supp Table 1**.

**P<0.05*

**ADDITIONAL FILE 1 TABLE S5.** Univariable and Multivariable Predictors for Combined Cardiovascular Endpoints of New-Onset Myocardial Infarction, Ischemic Stroke, Congestive Heart Failure and Cardiovascular Death.

|  | **Univariable Model** | | **Multivariable Model** ^2^ | | **Fully-Adjusted Model** ^3^ | | **Vascular Function Model** ^4^ | |
| --- | --- | --- | --- | --- | --- | --- | --- | --- |
|  | HR [95%CI] *^1^* | P-value | HR [95%CI] *^1^* | P-value | HR [95%CI] *^1^* | P-value | HR [95%CI] *^1^* | P-value |
| **Male** | 0.80 [0.50 to 1.27] | 0.80 |  |  | 1.91 [0.88 to 4.13] | 0.10 |  |  |
| **Age (years)** | 1.12 [[1.09 to 1.15] | *<0.001** | 1.09 [1.05 to 1.13] | *<0.001** | 1.09 [1.04 to 1.13] | *<0.001** | 1.09 [1.04 to 1.13] | *<0.001** |
| **Body mass index (kgm^-2^)** | 0.97 [0.91 to 1.03] | 0.97 |  |  | 0.95 [0.87 to 1.04] | 0.27 | 0.96 [0.87 to 1.05] | 0.37 |
| **Coronary Artery Disease** | 1.65 [1.06 to 2.57] | *0.026** | 1.34 [0.70 to 2.59] | 0.38 | 2.54 [1.08 to 5.98] | *0.033** | 2.54 [1.08 to 5.95] | *0.033** |
| **Prior stroke** | 1.25 [0.78 to 2.03] | 0.35 |  |  | 1.80 [0.81 to 4.00] | 0.15 | 1.80 [0.81 to 4.03] | 0.15 |
| **Diabetes mellitus** | 1.10 [0.72 to 1.68] | 0.66 |  |  | 0.97 [0.44 to 2.12] | 0.93 | 0.90 [0.40 to 2.00] | 0.79 |
| **Current/Past smoker** | 1.75 [1.14 to 2.69] | *0.011** | 1.18 [0.72 to 1.94] | 0.52 | 1.22 [0.66 to 2.27] | 0.53 | 1.18 [0.63 to 2.22] | 0.60 |
| **Regular Physical activity** | 0.76 [0.48 to 1.20] | 0.24 |  |  | 0.73 [0.40 to 1.34] | 0.31 | 0.76 [0.41 to 1.39] | 0.37 |
| **Resting pulse rate (bpm)** | 1.02 [1.01 to 1.03] | *0.001** | 1.00 [0.98 to 1.02] | 0.78 | 1.01 [0.98 to 1.03] | 0.67 | 1.00 [0.98 to 1.03] | 0.75 |
| **Systolic BP (mmHg)** | 1.02 [1.015 to 1.033] | *<0.001** | 1.01 [1.00 to 1.03] | 0.066 | 1.01 [0.99 to 1.03] | 0.38 | 1.01 [0.99 to 1.03] | 0.40 |
| **Diastolic BP (mmHg)** | 1.01 [0.99 to 1.03] | 0.57 |  |  | 1.00 [0.97 to 1.04] | 0.84 | 1.01 [0.97 to 1.05] | 0.81 |
| **LDL-cholesterol(mmol/L)** | 0.91 [0.68 to 1.23] | 0.55 |  |  | 1.09 [0.68 t 1.74] | 0.72 | 1.06 [0.66 to 1.71] | 0.80 |
| **HDL-cholesterol(mmol/L)** | 1.06 [0.57 to 2.00] | 0.85 |  |  | 2.23 [0.89 to 5.57] | 0.086 | 2.20 [0.85 to 5.64] | 0.10 |
| **Triglycerides (mmol/L)** | 0.81 [0.59 to 1.09] | 0.16 | 0.87 [0.61 to 1.24] | 0.44 | 0.86 [0.57 to 1.30] | 0.48 | 0.86 [0.57 to 1.30] | 0.48 |
| **Fasting glucose (mmol/L)** | 0.99 [0.88 to 1.11] | 0.79 |  |  | 0.95 [0.77 to 1.17] | 0.62 | 0.97 [0.78 to 1.20] | 0.75 |
| **HbA1c (%)** | 1.08 [0.93 to 1.26] | 0.30 |  |  | 1.28 [0.94 to 1.74] | 0.11 | 1.28 [0.94 to 1.75] | 0.12 |
| **hs-CRP (mg/L)** | 1.05 [1.03 to 1.07] | *<0.001** | 1.05 [1.03 to 1.07] | *<0.001** | 1.06 [1.03 to 1.09] | *<0.001** | 1.06 [1.03 to 10.9] | *<0.001** |
| **Serum creatinine (µmol/L)** | 1.014 [1.011 to 1.017] | *<0.001** | 1.005 [1.00 to 1.01] | 0.069 | 1.01 [1.00 to 1.02] | *0.002** | 1.01 [1.00 to 1.02] | *0.006** |
| **Medications:** |  |  |  |  |  |  |  |  |
| ACEI/ ARB | 1.71 [1.08 to 2.72] | *0.022** | 1.36 [0.78 to 2.36] | 0.28 | 1.64 [0.91 to 2.93] | 0.099 | 1.50 [0.82 to 2.74] | 0.19 |
| Beta-blockers | 1.41 [0.91 to 2.19] | 0.13 | 0.75 [0.44 to 1.27] | 0.28 | 0.90 [0.50 to 1.63] | 0.90 | 0.90 [0.50 to 1.62] | 0.73 |
| Calcium channel blockers | 1.36 [0.86 to 2.14] | 0.19 | 1.16 [0.64 to 2.08] | 0.63 | 1.35 [0.72 to 2.55] | 0.35 | 1.28 [0.67 to 2.44] | 0.45 |
| Aspirin | 1.65 [0.99 to 2.74] | 0.053 | 1.25 [0.64 to 2.44] | 0.51 | 1.37 [0.62 to 3.05] | 0.44 | 1.37 [0.61 to 3.06] | 0.45 |
| Statin | 1.28 [0.81 to 2.00] | 0.28 |  |  | 0.62 [0.29 to 1.31] | 0.21 | 0.67 [0.31 to 1.44] | 0.30 |
| **Widened QRS >120ms** | 2.17 [1.12 to 4.21] | *0.021** | 1.02 [0.47 to 2.22] | 0.96 | 0.94 [0.39 to 2.23] | 0.88 | 0.93 [0.39 to 2.23] | 0.88 |
| **Carotid IMT (mm)** | 4.17 [2.66 to 6.55] | *<0.001** |  |  |  |  | 1.51 [0.67 to 3.43] | 0.32 |
| **PR prolongation >200ms** | 2.45 [1.5 to 3.98] | *<0.001** | 1.95 [1.11 to 3.43] | *0.020** | 2.40 [1.30 to 4.43] | *0.005** | 2.33 [1.26 to 4.32] | *0.007** |

*^1^* Hazard ratio (HR) estimates and 95% confidence interval of combined cardiovascular endpoints predicted by variable of interest as derived from univariable and multivariable Cox regression;

^2^Adjusted for potential confounders as defined as from univariable analysis with p-value <0.20;

^3^ Adjusted for all potentially confounding variables as defined *a priori*.

^4^ Adjusted for all potentially confounding variables as defined *a priori* and vascular function marker (carotid IMT)

Abbreviations as **Supp Table 1**.

**P<0.05*

**ADDITIONAL FILE 1 TABLE S6. Estimates of Sensitivity (Se), Specificity (Sp), Positive Predictive Value (PPV) and Negative Predictive Value (NPV) of PR Interval in the Prediction for Cardiovascular (CV) Events.**

| **Event if**  **PR interval**  **≥ (ms)** | **CV death** | | | | **Ischemic stroke** | | | | **Myocardial Infarction** | | | | **Congestive Heart Failure** | | | | **Combined CV endpoints** | | | |
| --- | --- | --- | --- | --- | --- | --- | --- | --- | --- | --- | --- | --- | --- | --- | --- | --- | --- | --- | --- | --- |
|  | ***Se***  ***(%)*** | ***Sp***  ***(%)*** | ***PPV***  ***(%)*** | ***NPV (%)*** | ***Se***  ***(%)*** | ***Sp***  ***(%)*** | ***PPV***  ***(%)*** | ***NPV (%)*** | ***Se***  ***(%)*** | ***Sp***  ***(%)*** | ***PPV***  ***(%)*** | ***NPV (%)*** | ***Se***  ***(%)*** | ***Sp***  ***(%)*** | ***PPV***  ***(%)*** | ***NPV (%)*** | ***Se***  ***(%)*** | ***Sp***  ***(%)*** | ***PPV***  ***(%)*** | ***NPV (%)*** |
| 73 | 100 | 0 | 5.0 | - | 100 | 0 | 4.0 | - | 100 | 0 | 4.0 | - | 100 | 0 | 6.0 | - | 100 | 0 | 19.0 | - |
| 92 | 96.3 | 0 | 4.8 | 0.0 | 95.2 | 0 | 3.8 | 0.0 | 100 | 0.2 | 4.0 | 100.0 | 100 | 0.2 | 6.0 | 100.0 | 98.9 | 0 | 18.8 | 0.0 |
| 113 | 96.3 | 0.2 | 4.8 | 50.7 | 95.2 | 0.2 | 3.8 | 50.0 | 100 | 0.4 | 4.0 | 100.0 | 100 | 0.4 | 6.0 | 100.0 | 98.9 | 0.2 | 18.9 | 43.7 |
| 117 | 96.3 | 0.5 | 4.8 | 72.0 | 95.2 | 0.5 | 3.8 | 71.4 | 100 | 0.7 | 4.0 | 100.0 | 100 | 0.7 | 6.0 | 100.0 | 98.9 | 0.6 | 18.9 | 69.9 |
| 119 | 92.6 | 0.7 | 4.7 | 64.3 | 95.2 | 0.9 | 3.8 | 81.8 | 100 | 1.1 | 4.0 | 100.0 | 100 | 1.1 | 6.1 | 100.0 | 97.7 | 0.8 | 18.8 | 59.7 |
| 122 | 92.6 | 1.2 | 4.7 | 75.5 | 90.5 | 1.2 | 3.7 | 75.2 | 100 | 1.6 | 4.1 | 100.0 | 100 | 1.6 | 6.1 | 100.0 | 96.6 | 1.2 | 18.7 | 60.1 |
| 125 | 92.6 | 1.6 | 4.7 | 80.4 | 90.5 | 1.6 | 3.7 | 80.2 | 100 | 1.9 | 4.1 | 100.0 | 100 | 2 | 6.1 | 100.0 | 96.6 | 1.6 | 18.7 | 66.7 |
| 127 | 92.6 | 1.9 | 4.7 | 83.0 | 90.5 | 1.9 | 3.7 | 82.8 | 100 | 2.3 | 4.1 | 100.0 | 100 | 2.3 | 6.1 | 100.0 | 96.6 | 2 | 18.8 | 71.5 |
| 129 | 92.6 | 2.8 | 4.8 | 87.8 | 90.5 | 2.8 | 3.7 | 87.6 | 100 | 3.2 | 4.1 | 100.0 | 100 | 3.2 | 6.2 | 100.0 | 96.6 | 2.9 | 18.9 | 78.4 |
| 131 | 92.6 | 3.5 | 4.8 | 90.0 | 90.5 | 3.5 | 3.8 | 89.8 | 100 | 3.9 | 4.2 | 100.0 | 97.3 | 3.7 | 6.1 | 95.5 | 95.4 | 3.5 | 18.8 | 76.4 |
| 133 | 92.6 | 3.9 | 4.8 | 90.9 | 90.5 | 3.8 | 3.8 | 90.6 | 100 | 4.2 | 4.2 | 100.0 | 97.3 | 4.1 | 6.1 | 96.0 | 95.4 | 3.9 | 18.9 | 78.3 |
| 135 | 85.2 | 4.9 | 4.5 | 86.3 | 90.5 | 5.2 | 3.8 | 92.9 | 100 | 5.6 | 4.2 | 100.0 | 97.3 | 5.5 | 6.2 | 97.0 | 93.1 | 5.1 | 18.7 | 75.9 |
| 137 | 85.2 | 6.3 | 4.6 | 89.0 | 90.5 | 6.6 | 3.9 | 94.3 | 100 | 7 | 4.3 | 100.0 | 97.3 | 7 | 6.3 | 97.6 | 93.1 | 6.7 | 19.0 | 80.5 |
| 139 | 85.2 | 7.4 | 4.6 | 90.5 | 90.5 | 7.6 | 3.9 | 95.0 | 100 | 8.1 | 4.3 | 100.0 | 97.3 | 8 | 6.3 | 97.9 | 93.1 | 7.8 | 19.1 | 82.8 |
| 141 | 81.5 | 8.4 | 4.5 | 89.6 | 90.5 | 8.9 | 4.0 | 95.7 | 100 | 9.3 | 4.4 | 100.0 | 94.6 | 9.1 | 6.2 | 96.4 | 92 | 9 | 19.2 | 82.7 |
| 143 | 77.8 | 10.2 | 4.4 | 89.7 | 81 | 10.4 | 3.6 | 92.9 | 100 | 11.2 | 4.5 | 100.0 | 91.9 | 10.9 | 6.2 | 95.5 | 88.5 | 10.6 | 18.8 | 79.7 |
| 145 | 77.8 | 11.8 | 4.4 | 91.0 | 81 | 12 | 3.7 | 93.8 | 96.2 | 12.6 | 4.4 | 98.8 | 89.2 | 12.3 | 6.1 | 94.7 | 87.4 | 12.2 | 18.9 | 80.5 |
| 147 | 74.1 | 14.2 | 4.3 | 91.2 | 76.2 | 14.4 | 3.6 | 93.6 | 96.2 | 15.2 | 4.5 | 99.0 | 83.8 | 14.6 | 5.9 | 93.4 | 83.9 | 14.5 | 18.7 | 79.3 |
| 149 | 74.1 | 16.7 | 4.5 | 92.5 | 71.4 | 16.7 | 3.4 | 93.3 | 96.2 | 17.7 | 4.6 | 99.1 | 83.8 | 17.1 | 6.1 | 94.3 | 82.8 | 17.1 | 19.0 | 80.9 |
| 151 | 74.1 | 19.6 | 4.6 | 93.5 | 66.7 | 19.4 | 3.3 | 93.3 | 96.2 | 20.7 | 4.8 | 99.2 | 81.1 | 20 | 6.1 | 94.3 | 80.5 | 20 | 19.1 | 81.4 |
| 153 | 74.1 | 23.7 | 4.9 | 94.6 | 66.7 | 23.4 | 3.5 | 94.4 | 92.3 | 24.5 | 4.8 | 98.7 | 81.1 | 24.1 | 6.4 | 95.2 | 79.3 | 24.3 | 19.7 | 83.3 |
| 155 | 70.4 | 27.2 | 4.8 | 94.6 | 61.9 | 26.9 | 3.4 | 94.4 | 92.3 | 28.2 | 5.1 | 98.9 | 78.4 | 27.7 | 6.5 | 95.3 | 75.9 | 27.8 | 19.8 | 83.1 |
| 157 | 70.4 | 29.3 | 5.0 | 95.0 | 61.9 | 29 | 3.5 | 94.8 | 92.3 | 30.3 | 5.2 | 99.0 | 78.4 | 29.8 | 6.7 | 95.6 | 75.9 | 30.2 | 20.3 | 84.2 |
| 158.5 | 70.4 | 32.1 | 5.2 | 95.4 | 61.9 | 31.8 | 3.6 | 95.2 | 92.3 | 33.1 | 5.4 | 99.0 | 75.7 | 32.5 | 6.7 | 95.4 | 74.7 | 33.1 | 20.8 | 84.8 |
| 159.5 | 70.4 | 32.3 | 5.2 | 95.4 | 61.9 | 31.9 | 3.6 | 95.3 | 92.3 | 33.3 | 5.5 | 99.0 | 75.7 | 32.7 | 6.7 | 95.5 | 74.7 | 33.3 | 20.8 | 84.9 |
| 161 | 70.4 | 36.5 | 5.5 | 95.9 | 57.1 | 35.9 | 3.6 | 95.3 | 88.5 | 37.3 | 5.6 | 98.7 | 70.3 | 36.6 | 6.6 | 95.1 | 71.3 | 37.5 | 21.1 | 84.8 |
| 163 | 70.4 | 40.9 | 5.9 | 96.3 | 52.4 | 40.1 | 3.5 | 95.3 | 84.6 | 41.5 | 5.7 | 98.5 | 70.3 | 41.1 | 7.1 | 95.6 | 69 | 42 | 21.8 | 85.2 |
| 165 | 70.4 | 44.9 | 6.3 | 96.6 | 52.4 | 44.1 | 3.8 | 95.7 | 84.6 | 45.5 | 6.1 | 98.6 | 70.3 | 45.2 | 7.6 | 96.0 | 69 | 46.5 | 23.2 | 86.5 |
| 167 | 70.4 | 49.3 | 6.8 | 96.9 | 42.9 | 48.1 | 3.3 | 95.3 | 80.8 | 49.7 | 6.3 | 98.4 | 67.6 | 49.5 | 7.9 | 96.0 | 65.5 | 50.8 | 23.8 | 86.3 |
| 169 | 70.4 | 51.9 | 7.2 | 97.1 | 42.9 | 50.7 | 3.5 | 95.5 | 80.8 | 52.4 | 6.6 | 98.5 | 64.9 | 52 | 7.9 | 95.9 | 64.4 | 53.5 | 24.5 | 86.5 |
| 171 | 70.4 | 55.3 | 7.7 | 97.3 | 42.9 | 54 | 3.7 | 95.8 | 80.8 | 55.7 | 7.1 | 98.6 | 62.2 | 55.2 | 8.1 | 95.8 | 63.2 | 57.1 | 25.7 | 86.9 |
| 173 | 70.4 | 58.2 | 8.1 | 97.4 | 42.9 | 56.9 | 4.0 | 96.0 | 76.9 | 58.5 | 7.2 | 98.4 | 62.2 | 58.2 | 8.7 | 96.0 | 62.1 | 60.2 | 26.8 | 87.1 |
| 175 | 70.4 | 61.6 | 8.8 | 97.5 | 38.1 | 60.1 | 3.8 | 95.9 | 69.2 | 61.5 | 7.0 | 98.0 | 56.8 | 61.2 | 8.5 | 95.7 | 57.5 | 63.1 | 26.8 | 86.4 |
| 177 | 66.7 | 64.9 | 9.1 | 97.4 | 38.1 | 63.5 | 4.2 | 96.1 | 61.5 | 64.6 | 6.8 | 97.6 | 48.6 | 64.3 | 8.0 | 95.1 | 52.9 | 66.3 | 26.9 | 85.7 |
| 179 | 66.7 | 67.7 | 9.8 | 97.5 | 38.1 | 66.3 | 4.5 | 96.3 | 61.5 | 67.4 | 7.3 | 97.7 | 45.9 | 67 | 8.2 | 95.1 | 51.7 | 69.2 | 28.3 | 85.9 |
| 181 | 63 | 69.8 | 9.9 | 97.3 | 38.1 | 68.6 | 4.8 | 96.4 | 61.5 | 69.7 | 7.8 | 97.8 | 43.2 | 69.1 | 8.2 | 95.0 | 49.4 | 71.4 | 28.8 | 85.7 |
| 183 | 59.3 | 72.3 | 10.1 | 97.1 | 38.1 | 71.2 | 5.2 | 96.5 | 57.7 | 72.2 | 8.0 | 97.6 | 43.2 | 71.8 | 8.9 | 95.2 | 47.1 | 73.9 | 29.7 | 85.6 |
| 185 | 55.6 | 74.4 | 10.3 | 97.0 | 38.1 | 73.4 | 5.6 | 96.6 | 53.8 | 74.3 | 8.0 | 97.5 | 43.2 | 74.1 | 9.6 | 95.3 | 44.8 | 76.1 | 30.5 | 85.5 |
| 187 | 55.6 | 76.1 | 10.9 | 97.0 | 38.1 | 75.2 | 6.0 | 96.7 | 50 | 75.8 | 7.9 | 97.3 | 43.2 | 75.9 | 10.3 | 95.4 | 43.7 | 77.8 | 31.6 | 85.5 |
| 189 | 55.6 | 78.1 | 11.8 | 97.1 | 38.1 | 77.1 | 6.5 | 96.8 | 46.2 | 77.6 | 7.9 | 97.2 | 40.5 | 77.7 | 10.4 | 95.3 | 41.4 | 79.6 | 32.3 | 85.3 |
| 191 | 55.6 | 80.7 | 13.2 | 97.2 | 38.1 | 79.7 | 7.3 | 96.9 | 34.6 | 79.7 | 6.6 | 96.7 | 37.8 | 80.2 | 10.9 | 95.3 | 37.9 | 82 | 33.1 | 84.9 |
| 193 | 48.1 | 81.9 | 12.3 | 96.8 | 38.1 | 81.2 | 7.8 | 96.9 | 34.6 | 81.3 | 7.2 | 96.8 | 37.8 | 81.8 | 11.7 | 95.4 | 35.6 | 83.3 | 33.3 | 84.6 |
| 195 | 44.4 | 83.2 | 12.2 | 96.6 | 38.1 | 82.6 | 8.4 | 97.0 | 30.8 | 82.5 | 6.8 | 96.6 | 37.8 | 83.2 | 12.6 | 95.4 | 33.3 | 84.5 | 33.5 | 84.4 |
| 197 | 44.4 | 84.6 | 13.2 | 96.7 | 33.3 | 83.9 | 7.9 | 96.8 | 30.8 | 83.9 | 7.4 | 96.7 | 32.4 | 84.3 | 11.6 | 95.1 | 31 | 85.7 | 33.7 | 84.1 |
| 199 | 40.7 | 86.1 | 13.4 | 96.5 | 33.3 | 85.6 | 8.8 | 96.9 | 26.9 | 85.5 | 7.2 | 96.6 | 32.4 | 86.1 | 13.0 | 95.2 | 29.9 | 87.5 | 35.9 | 84.2 |
| 201 | 37 | 87.9 | 13.9 | 96.4 | 33.3 | 87.5 | 10.0 | 96.9 | 23.1 | 87.2 | 7.0 | 96.5 | 24.3 | 87.5 | 11.0 | 94.8 | 25.3 | 88.8 | 34.6 | 83.5 |
| 203 | 37 | 89.1 | 15.2 | 96.4 | 33.3 | 88.7 | 10.9 | 97.0 | 23.1 | 88.4 | 7.7 | 96.5 | 21.6 | 88.6 | 10.8 | 94.7 | 24.1 | 90 | 36.1 | 83.5 |
| 205 | 37 | 89.6 | 15.8 | 96.4 | 33.3 | 89.2 | 11.4 | 97.0 | 23.1 | 89 | 8.0 | 96.5 | 21.6 | 89.1 | 11.2 | 94.7 | 24.1 | 90.6 | 37.6 | 83.6 |
| 207 | 33.3 | 90.9 | 16.1 | 96.3 | 33.3 | 90.6 | 12.9 | 97.0 | 19.2 | 90.2 | 7.5 | 96.4 | 16.2 | 90.2 | 9.5 | 94.4 | 20.7 | 91.6 | 36.6 | 83.1 |
| 209 | 29.6 | 91.9 | 16.1 | 96.1 | 33.3 | 91.8 | 14.5 | 97.1 | 15.4 | 91.2 | 6.8 | 96.3 | 16.2 | 91.4 | 10.7 | 94.5 | 18.4 | 92.5 | 36.5 | 82.9 |
| 211 | 22.2 | 92.8 | 14.0 | 95.8 | 19 | 92.5 | 9.5 | 96.5 | 11.5 | 92.3 | 5.9 | 96.2 | 13.5 | 92.5 | 10.3 | 94.4 | 14.9 | 93.3 | 34.3 | 82.4 |
| 213 | 22.2 | 93.2 | 14.7 | 95.8 | 14.3 | 92.7 | 7.5 | 96.3 | 11.5 | 92.6 | 6.1 | 96.2 | 13.5 | 92.9 | 10.8 | 94.4 | 13.8 | 93.5 | 33.2 | 82.2 |
| 215 | 22.2 | 93.5 | 15.2 | 95.8 | 9.5 | 92.9 | 5.3 | 96.1 | 11.5 | 93 | 6.4 | 96.2 | 13.5 | 93.2 | 11.2 | 94.4 | 12.6 | 93.7 | 31.9 | 82.0 |
| 217 | 22.2 | 93.7 | 15.6 | 95.8 | 9.5 | 93.1 | 5.4 | 96.1 | 11.5 | 93.2 | 6.6 | 96.2 | 13.5 | 93.4 | 11.5 | 94.4 | 12.6 | 93.9 | 32.6 | 82.1 |
| 219 | 22.2 | 94.2 | 16.8 | 95.8 | 9.5 | 93.6 | 5.8 | 96.1 | 11.5 | 93.7 | 7.1 | 96.2 | 13.5 | 93.9 | 12.4 | 94.4 | 12.6 | 94.5 | 35.0 | 82.2 |
| 221 | 14.8 | 94.7 | 12.8 | 95.5 | 9.5 | 94.4 | 6.6 | 96.2 | 11.5 | 94.6 | 8.2 | 96.2 | 8.1 | 94.5 | 8.6 | 94.2 | 9.2 | 94.9 | 29.7 | 81.7 |
| 223 | 14.8 | 95.3 | 14.2 | 95.5 | 9.5 | 95 | 7.3 | 96.2 | 11.5 | 95.1 | 8.9 | 96.3 | 8.1 | 95 | 9.4 | 94.2 | 9.2 | 95.5 | 32.4 | 81.8 |
| 225 | 14.8 | 96.1 | 16.6 | 95.5 | 4.8 | 95.7 | 4.4 | 96.0 | 7.7 | 95.8 | 7.1 | 96.1 | 2.7 | 95.5 | 3.7 | 93.9 | 6.9 | 96.1 | 29.3 | 81.5 |
| 227 | 14.8 | 96.3 | 17.4 | 95.6 | 4.8 | 95.8 | 4.5 | 96.0 | 7.7 | 96 | 7.4 | 96.1 | 2.7 | 95.7 | 3.9 | 93.9 | 6.9 | 96.3 | 30.4 | 81.5 |
| 229 | 14.8 | 96.8 | 19.6 | 95.6 | 4.8 | 96.4 | 5.3 | 96.0 | 7.7 | 96.5 | 8.4 | 96.2 | 2.7 | 96.2 | 4.3 | 93.9 | 6.9 | 96.9 | 34.3 | 81.6 |
| 231 | 14.8 | 97.2 | 21.8 | 95.6 | 0 | 96.5 | 0.0 | 95.9 | 7.7 | 96.8 | 9.1 | 96.2 | 2.7 | 96.6 | 4.8 | 94.0 | 5.7 | 97.1 | 31.6 | 81.4 |
| 233 | 11.1 | 98.2 | 24.5 | 95.5 | 0 | 97.7 | 0.0 | 95.9 | 7.7 | 98.1 | 14.4 | 96.2 | 2.7 | 97.9 | 7.6 | 94.0 | 4.6 | 98.2 | 37.5 | 81.4 |
| 235 | 11.1 | 98.6 | 29.4 | 95.5 | 0 | 98.1 | 0.0 | 95.9 | 7.7 | 98.4 | 16.7 | 96.2 | 2.7 | 98.2 | 8.7 | 94.1 | 4.6 | 98.6 | 43.5 | 81.5 |
| 239 | 7.4 | 98.6 | 21.8 | 95.3 | 0 | 98.3 | 0.0 | 95.9 | 3.8 | 98.4 | 9.0 | 96.1 | 0 | 98.2 | 0.0 | 93.9 | 3.4 | 98.6 | 36.3 | 81.3 |
| 244 | 7.4 | 98.8 | 24.5 | 95.3 | 0 | 98.4 | 0.0 | 95.9 | 3.8 | 98.6 | 10.2 | 96.1 | 0 | 98.4 | 0.0 | 93.9 | 3.4 | 98.8 | 39.9 | 81.3 |
| 249 | 3.7 | 98.9 | 15.0 | 95.1 | 0 | 98.8 | 0.0 | 96.0 | 3.8 | 98.9 | 12.6 | 96.1 | 0 | 98.7 | 0.0 | 93.9 | 2.3 | 99 | 35.0 | 81.2 |
| 254 | 3.7 | 99.1 | 17.8 | 95.1 | 0 | 99 | 0.0 | 96.0 | 3.8 | 99.1 | 15.0 | 96.1 | 0 | 98.9 | 0.0 | 93.9 | 2.3 | 99.2 | 40.3 | 81.2 |
| 258 | 3.7 | 99.3 | 21.8 | 95.1 | 0 | 99.1 | 0.0 | 96.0 | 3.8 | 99.3 | 18.4 | 96.1 | 0 | 99.1 | 0.0 | 93.9 | 2.3 | 99.4 | 47.3 | 81.3 |
| 268 | 0 | 99.3 | 0.0 | 95.0 | 0 | 99.3 | 0.0 | 96.0 | 3.8 | 99.5 | 24.1 | 96.1 | 0 | 99.3 | 0.0 | 94.0 | 1.1 | 99.4 | 30.1 | 81.1 |
| 279 | 0 | 99.5 | 0.0 | 95.0 | 0 | 99.5 | 0.0 | 96.0 | 3.8 | 99.6 | 28.4 | 96.1 | 0 | 99.5 | 0.0 | 94.0 | 1.1 | 99.6 | 39.2 | 81.1 |
| 292 | 0 | 99.6 | 0.0 | 95.0 | 0 | 99.7 | 0.0 | 96.0 | 0 | 99.6 | 0.0 | 96.0 | 0 | 99.6 | 0.0 | 94.0 | 0 | 99.6 | 0.0 | 80.9 |
| 311 | 0 | 99.8 | 0.0 | 95.0 | 0 | 99.8 | 0.0 | 96.0 | 0 | 99.8 | 0.0 | 96.0 | 0 | 99.8 | 0.0 | 94.0 | 0 | 99.8 | 0.0 | 81.0 |
| 321 | 0 | 100 | - | 95.0 | 0 | 100 | - | 96.0 | 0 | 100 | - | 96.0 | 0 | 100 | - | 94.0 | 0 | 100 | - | 81.0 |
